# Clusters from chronic conditions in the Danish adult population

**DOI:** 10.1371/journal.pone.0302535

**Published:** 2024-04-30

**Authors:** Anders Stockmarr, Anne Frølich

**Affiliations:** 1 Department of Applied Mathematics and Computer Science, Technical University of Denmark, Lyngby, Denmark; 2 Innovation and Research Centre for Multimorbidity, Slagelse Hospital, Slagelse, Denmark; 3 Department of Public Health, University of Copenhagen, Copenhagen, Denmark; Faculty of Health Sciences - Universidade da Beira Interior, PORTUGAL

## Abstract

Multimorbidity, the presence of 2 or more chronic conditions in a person at the same time, is an increasing public health concern, which affects individuals through reduced health related quality of life, and society through increased need for healthcare services. Yet the structure of chronic conditions in individuals with multimorbidity, viewed as a population, is largely unmapped. We use algorithmic diagnoses and the K-means algorithm to cluster the entire 2015 Danish multimorbidity population into 5 clusters. The study introduces the concept of rim data as an additional tool for determining the number of clusters. We label the 5 clusters the Allergies, Chronic Heart Conditions, Diabetes, Hypercholesterolemia, and Musculoskeletal and Psychiatric Conditions clusters, and demonstrate that for 99.32% of the population, the cluster allocation can be determined from the diagnoses of 4–5 conditions. Clusters are characterized through most prevalent conditions, absent conditions, over- or under-represented conditions, and co-occurrence of conditions. Clusters are further characterized through socioeconomic variables and healthcare service utilizations. Additionally, geographical variations throughout Denmark are studied at the regional and municipality level. We find that subdivision into municipality levels suggests that the Allergies cluster frequency is positively associated with socioeconomic status, while the subdivision suggests that frequencies for clusters Diabetes and Hypercholesterolemia are negatively correlated with socioeconomic status. We detect no indication of association to socioeconomic status for the Chronic Heart Conditions cluster and the Musculoskeletal and Psychiatric Conditions cluster. Additional spatial variation is revealed, some of which may be related to urban/rural populations. Our work constitutes a step in the process of characterizing multimorbidity populations, leading to increased comprehension of the nature of multimorbidity, and towards potential applications to individual-based care, prevention, the development of clinical guidelines, and population management.

## Introduction

Multimorbidity is increasingly recognized as a worldwide, serious public health concern [1]. The prevalence rates are increasing due to the changing demography of aging populations and also increasingly better health technologies [2]. The burden of multimorbidity varies across ages, with the highest prevalence at the older ages, and the highest numbers in middle aged people [2–4]. Several risk factors for developing multimorbidity are well-known and described in the literature, including age, gender, socioeconomic status, lifestyle factors such as smoking, alcohol, low physical activity, and obesity [5–9]. Multimorbidity influence health outcomes and prognosis of illness, complications, and health related quality of life. The definition is ambiguous, and obviously dependent on the context [10]. WHO defines multimorbidity as “the coexistence of two or more chronic conditions in the same individual” [2]; but if one looks at the chronic conditions that each individual has, which we term the individual’s condition portfolio, among two different sets of chronic conditions, an individual may have multimorbidity for one set of chronic conditions and not for another. However, comparative studies indicate that the practical implications of this ambiguity are likely minor [3–4]. Ofori-Asenso R et al [11] uses 3 and 5 chronic conditions as thresholds for multimorbidity. In the present work, we use the WHO threshold of two or more chronic conditions in the same individual as the definition of multimorbidity.

Managing individuals with multimorbidity is a challenge facing health systems across the globe [12–15]. Developing effective clinical guidelines to direct high-quality care provision is challenging as the knowledge base for treatment of more chronic conditions in the same individual is low [16]. National Institute for Healthcare Excellence (NICE) in the UK has developed a guideline at the national level in 2016 that focus much on organization of care and patient centeredness in patients with multimorbidity [17]. Several problems with managing individuals with multimorbidity are well-described, among them are polypharmacy, multiple general practitioner (GP) and out-patient visits, more hospitalizations, longer hospital stays, and fragmented patient pathways [18–20]. To support effective care management, it is vital to clarify and map the structure and content of the condition portfolios, to be able to meet different needs in the various population segments [21]. Individual clinical management plans require clinical skills to be effective according to the conditions of the individuals. This is often challenging for individuals with complex needs [17], both because of the complexity of the individual needs, and because there are large numbers of different condition portfolios. However, if multimorbid individuals with condition portfolios that resemble each other are similar in terms of medical needs, then clinical guidelines for common condition portfolios should be developed. Further, one may conjecture that the same clinical expertise in specialists should be applicable for those groups of individuals. One way to assess similarity of condition portfolios is through cluster analysis of individuals’ conditions, where individuals with similar condition portfolios are grouped into the same cluster.

The aim of this study was to identify and describe clusters of individuals with multimorbidity based on their chronic conditions using the K-means method [22], and to discuss the cluster structure and potential applications in clinical settings. We characterized the clusters by the three most prevalent conditions and conditions not present in the cluster, over- and underrepresented conditions, the most common concomitant conditions, socioeconomic characteristics, and utilization of healthcare services.

The cluster characteristics may be informative for both population-based care in populations suffering from multimorbidity, the development of clinical guidelines for those populations, in individual-based care and for hypothesis generation on possible important disease mechanisms of factors important for cluster composition, progression of multimorbidity and prevention.

## Methods

### Data

The data used in this study originates from a cross sectional design study of all individuals aged 18 years and older who lived in Denmark on January 1^st^ in year 2015, counting 4.489.821 individuals.

Information about chronic conditions, socioeconomic characteristics (age, gender, educational attainment and occupation) and utilization of healthcare services (hospitalizations, bed days, out-patient visits), were extracted per January 1^st^, 2015, from national registers: The Danish National Patient Registry [23], the Danish Psychiatric Central Research Register [24], the Danish National Prescription Registry [25], the Danish National Health Service Registry [26], and the Danish Population Education Register [27].

National registers do not comprise direct information about the type of chronic conditions diagnosed in the primary sector. To ensure that we have information on chronic conditions for the total population, we used diagnostic algorithms developed by the Research Center for Prevention and Health at Glostrup University Hospital for 16 selected chronic conditions, using information from registers including data from both primary and secondary healthcare sectors [4, 28]. The chronic conditions were allergies, anxiety, back pain, cancer, chronic heart condition (CHC), chronic obstructive pulmonary disease (COPD), dementia, long term use of antidepressants (depression), diabetes, hypercholesterolemia, hypertension, osteoarthritis, osteoporosis, rheumatoid arthritis, schizophrenia, and stroke (Table 1). The term ‘conditions’ used in the following refers to the outcomes of the diagnostic algorithms, and the term ‘condition portfolio’ refers in the following to the collection of individual conditions that an individual has, as outcome of the algorithmic diagnoses. Among the 4.489.821 individuals considered, 2.394.292 (53.33%) individuals had no conditions, 2.095.529 (46.67%) individuals had at least one condition, of which 958.457 (45.74%) had exactly one condition, while 1.137.072 (54.26%) had more than one condition (multimorbidity). The essential algorithms for constructing the algorithmic diagnoses are published elsewhere [18, 29]. However, the application here is slightly different. The exact algorithms are listed in S1 Table.

**Table 1:** Numbers and prevalence rates of 16 chronic conditions in the multimorbidity population, at a national level and in the five Regions of Denmark. Chronic heart conditions (CHC), chronic obstructive pulmonary disease (COPD), hypercholesterolemia (hyperchol.), rheumatoid arthritis (rh. arthritis). Data for 2015.

Chronic Condition	Denmark n (%)	Capital Region of Denmark n (%)	Central Denmark Region n (%)	North Denmark Region n (%)	Region of Southern Denmark n (%)	Region Zealand n (%)
Allergies	301.614 (26.53)	90.681 (29.45)	66.028 (25.74)	31.808 (25.10)	66.418 (24.64)	46.679 (26.48)
Anxiety	2.324 (0.20)	567 (0.18)	692 (0.27)	184 (0.15)	604 (0.22)	277 (0.16)
Back pain	131.847 (11.60)	34.042 (11.06)	28.546 (11.13)	13.005 (10.26)	39.667 (14.71)	16.587 (9.41)
Cancer	117.194 (10.31)	34.528 (11.21)	24.093 (9.39)	12.588 (9.93)	26.989 (10.01)	18.996 (10.78)
CHC	205.678 (18.09)	57.214 (18.58)	48.064 (18.73)	21.588 (17.03)	44.432 (16.48)	34.380 (19.50)
COPD	205.052 (18.03)	58.760 (19.09)	45.241 (17.63)	22.243 (17.55)	46.581 (17.28)	32.227 (18.28)
Dementia	30.702 (2.70)	9.367 (3.04)	5.977 (2.33)	2.834 (2.24)	8.171 (3.03)	4.353 (2.47)
Depression	201.173 (17.69)	49.520 (16.08)	50.458 (19.67)	21.760 (17.17)	40.365 (18.44)	29.728 (16.86)
Diabetes	224.990 (19.79)	63.672 (20.68)	48.491 (18.90)	24.756 (19.53)	50.416 (18.70)	37.655 (21.36)
Hyperchol.	625.298 (54.99)	163.796 (53.20)	144.813 (56.44)	73.405 (57.92)	148.204 (54.97)	95.080 (53.93)
Hypertension	832.446 (73.21)	219.094 (71.16)	185.893 (72.45)	97.784 (77.16)	198.410 (73.60)	131.265 (74.46)
Osteoarthritis	160.665 (14.13)	45.781 (14.87)	36.104 (14.07)	14.771 (11.66)	40.365 (14.97)	23.644 (13.41)
Osteoporosis	170.904 (15.03)	45.570 (14.80)	40.879 (15.93)	18.236 (14.39)	43.143 (16.00)	23.076 (13.09)
Rh. arthritis	30.842 (2.71)	7.906 (2.57)	6.957 (2.71)	3.406 (2.69)	7.401 (2.75)	5.172 (2.93)
Schizophrenia	40.753 (3.58)	11.887 (3.86)	8.835 (3.44)	4.556 (3.59)	9.275 (3.44)	6.200 (3.52)
Stroke	95.350 (8.39)	27.236 (8.85)	19.694 (7.68)	9.421 (7.43)	22.699 (8.42)	16.300 (9.25)

The study population that we analyze in the following is comprised of the 1.137.072 multimorbid individuals in Denmark in 2015, relative to the 16 listed chronic conditions. Supplementary and comparative studies were performed on the larger population of individuals that had one or more chronic conditions.

We use spatial data for visualization from the Agency for Data Supply and Infrastructure, subject to the CC BY 4.0 license, see https://dataforsyningen.dk/asset/PDF/rettigheder_vilkaar/Vilk%C3%A5r%20for%20brug%20af%20frie%20geografiske%20data.pdf (in Danish).

### Statistical methods for portfolio clusters

**Fig 1:**
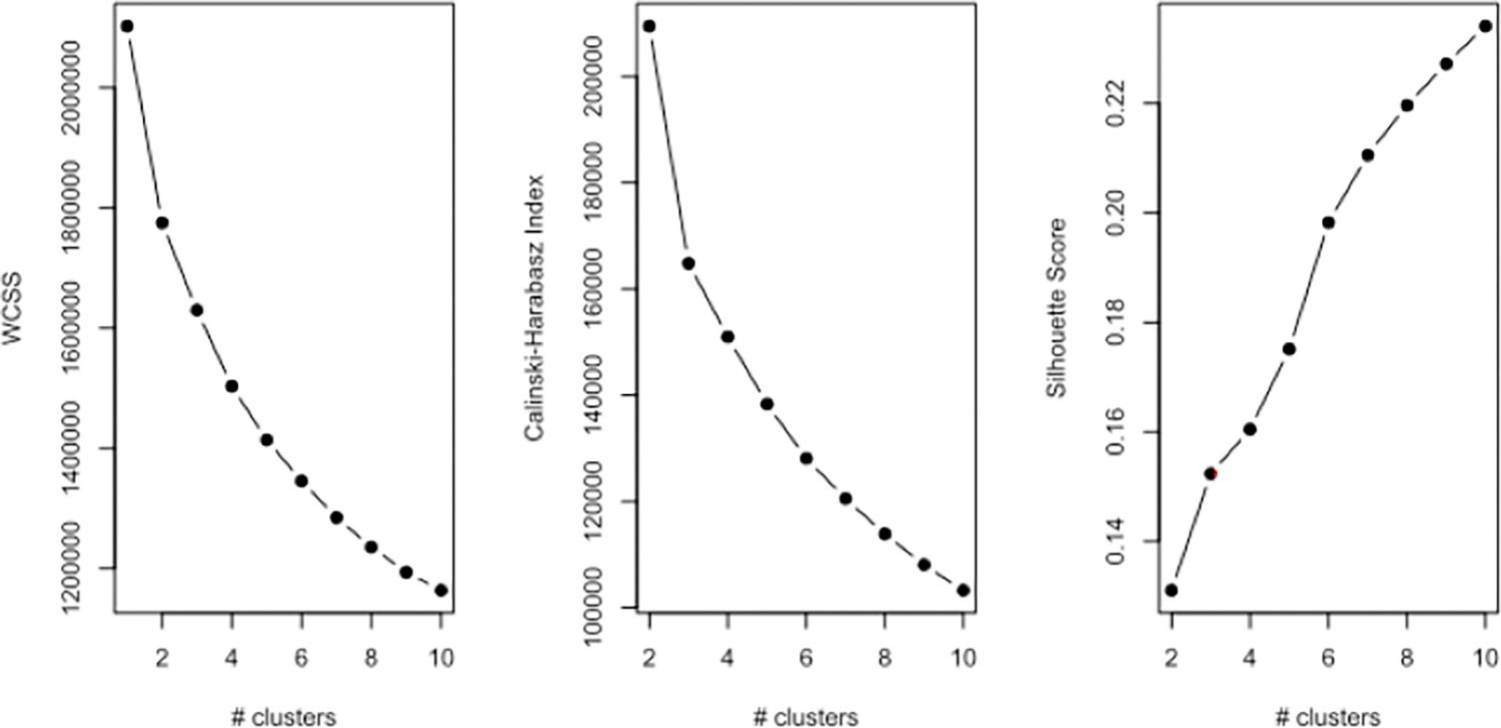
Within cluster sum of squares, Caliński-Harabasz Index and Silhouette score. The elbow of the WCSS and Caliński-Harabasz Index graphs point towards 3–6 clusters.

Condition portfolios for the study population were clustered with the K-means method using the Hartigan-Wong algorithm [30], varying the number of clusters between 1 and 10. To avoid impact from random initial configurations when applying the K-means method [31], 25 initial configurations were used. 200 runs of K-means were applied for each number of clusters. The minimum of the 200 within cluster sum of squares (WCSS) were recorded, and an optimal configuration was declared if the exact minimum value appeared more than 10 times out of the 200. If no optimal configuration was declared, another 200 runs of K-means were performed, and the configuration corresponding to the overall minimum was used as the optimal configuration. The minimum WCSS were displayed as a function of the number of clusters and used to support determination of the optimal number of clusters through the elbow method (Fig 1), supplemented with the Caliński-Harabasz index [32], the silhouette score [33] and rim data frequencies as described below. A similar procedure was applied to the population with one or more chronic diseases for comparison.

**Fig 2:**
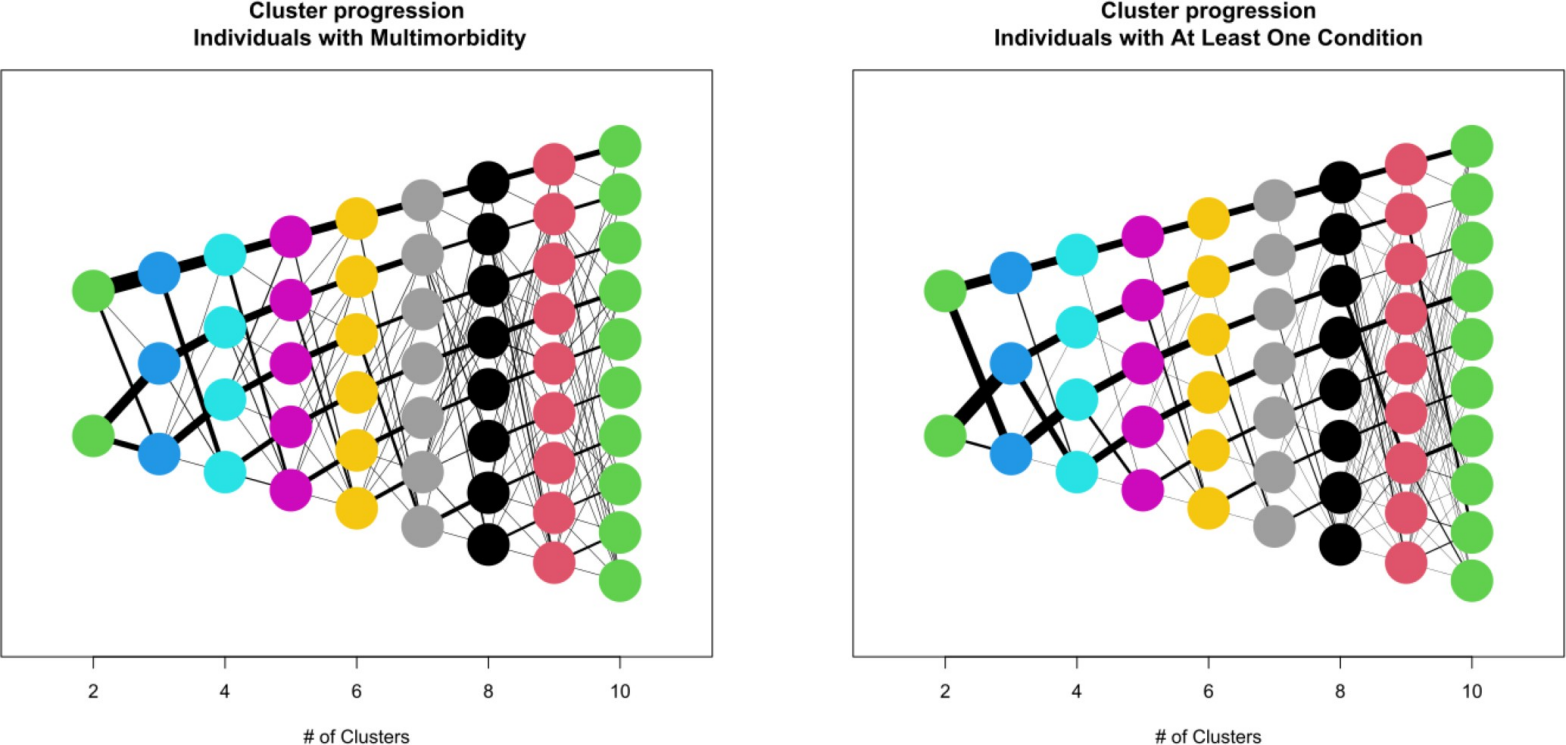
The progression between clusters when number of clusters vary from 2 to 10.

To illustrate the progression when the number of clusters is increased, and to support the optimal choice of number of clusters, we paired clusters to the previous set of clusters when increasing the number of clusters from k to k+1, for k = 1 to 9, as follows. We selected the configuration of k clusters among the new k+1 clusters where the sum of the Euclidian distances from the centroids of these to the centroids of the old k clusters was at a minimum. In this way, k clusters are paired to the previous set of clusters, while the one remaining cluster is termed the ‘new cluster’. The progression from 2 to 10 clusters was depicted graphically (Fig 2). Individuals where an increase in number of clusters meant assigning a cluster to this individual out of k+1 that was not paired to the individuals’ assigned cluster out of k, and was not the new cluster, were termed *rim data* for k clusters. Rim data are undesirable, as they indicate erractic cluster allocation, and in contrast to the WCSS, the Caliński-Harabasz index and the silhouette score, the rim data frequencies involve information from two neighboring clusterings, rather than one clustering.

### Rim data

**Fig 3:**
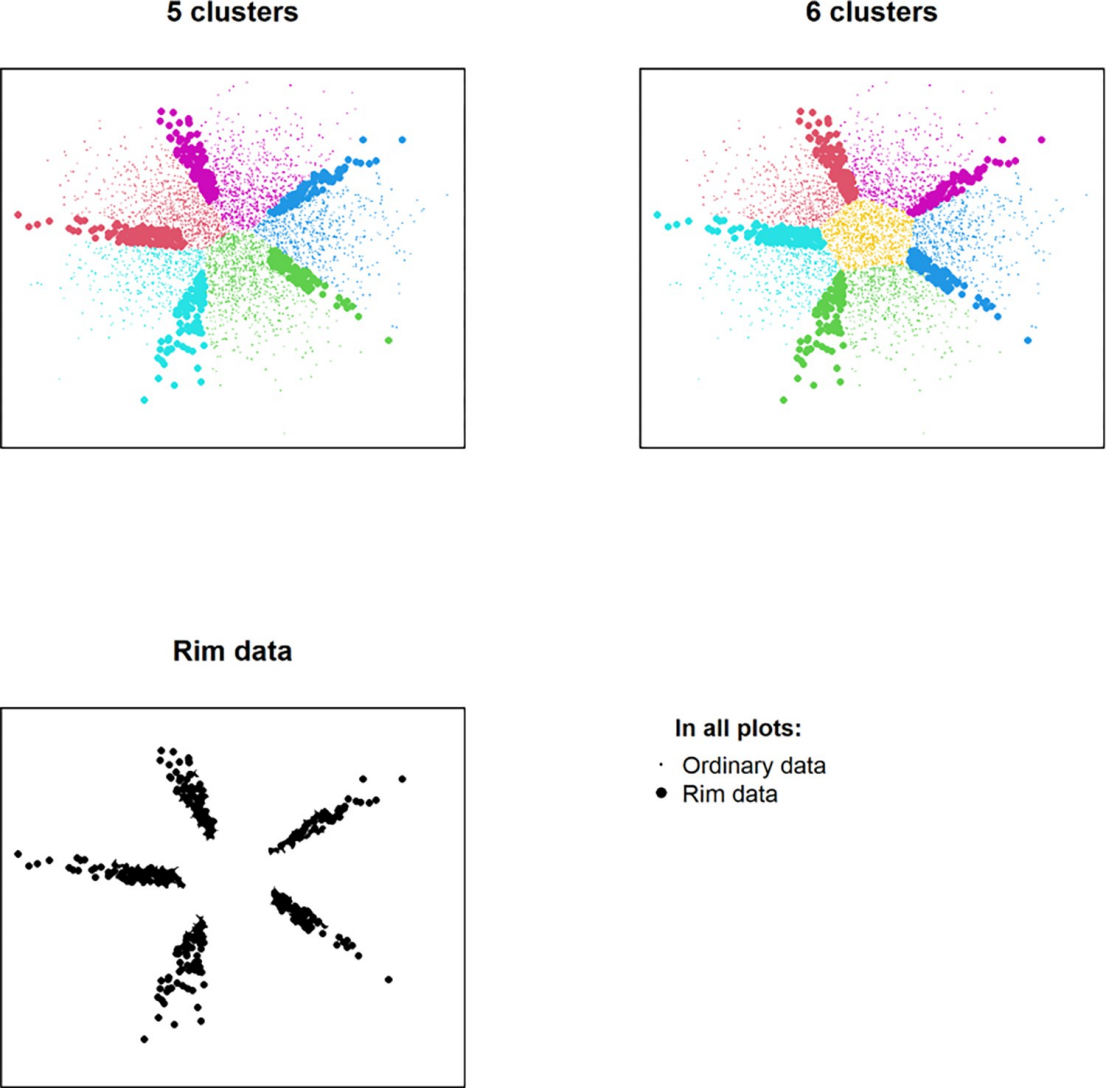
The position of simulated data in the plane, colored according to K-means clustering with 5 respectively 6 clusters. The new cluster appears in the center; the last figure shows rim data where erratic cluster allocation changes take place.

Pairing and rim data were illustrated with a case of 2-dimensional clustering (rather than our 16-dimensional) (Fig 3), indicating that the cluster configuration may change erratically for these data when the number of clusters is changed. In Fig 3, when increasing the amounts of clusters from 5 to 6, a ‘new cluster’, in the sense described above, appears in the middle of the subfigure to the upper right. The remaining 5 clusters are largely continuations of the clusters when the number of clusters is specified to 5, the subfigure to the upper left. However, it also appears that a slight clockwise rotation occurs in the cluster formation. In Fig 3, rim data are depicted with a larger font than the ordinary data, and the rotation is exemplified in t. ex. the large font points in the lower right of both top figures, which for 5 clusters are part of the green cluster, but for 6 clusters are part of the blue cluster. Thus, even though the 5 clusters are largely continued, disregarding their contribution to the ‘new cluster’, the rims of the clusters are slightly perturbed, causing points to erratically change cluster when the amount is increased from 5 to 6. All points with this erratic behavior, the rim data, are depicted in the lower left of Fig 3. It is clear from the figure that all of these points appear at the rim of the clusters, both when the numbers of clusters are 5 and 6.

All analyses were made with R version 4.3.1 [34].

### Ethical considerations

The Danish national registries are protected by the Danish Data Protection Act and can only be accessed following application and subsequent approval. No informed consent nor approval from the Danish Research Ethics Committees were needed for this study, since only national register data was used. Data are stored on secured servers at Statistics Denmark and were made available for research on August 31^st^, 2017. While information on the servers at Statistics Denmark in principle makes person identification possible, it is not permitted to extract data that may be used for such from the secured servers. Appropriate control measures are enforced, including a lower limit on the size of groups that may be averaged.

## Results

### Number of clusters

The elbow graph of the WCSS and the Caliński-Harabasz index both indicated that the relevant choice of number of clusters within a clinically relevant size (≤10) is around five (Fig 1). However, the graphs in Fig 1 do not depict a clear ‘elbow’, which suggests that an optimal number of clusters is far beyond the set limits for a clinically relevant number of clusters. This is supported by investigations of the silhouette score (Fig 1), which increases with the number of clusters up to 10. To further support the decision on the relevant number of clusters, we considered the progression in clusters illustrated in Fig 2 in terms of rim data.

**Fig 4:**
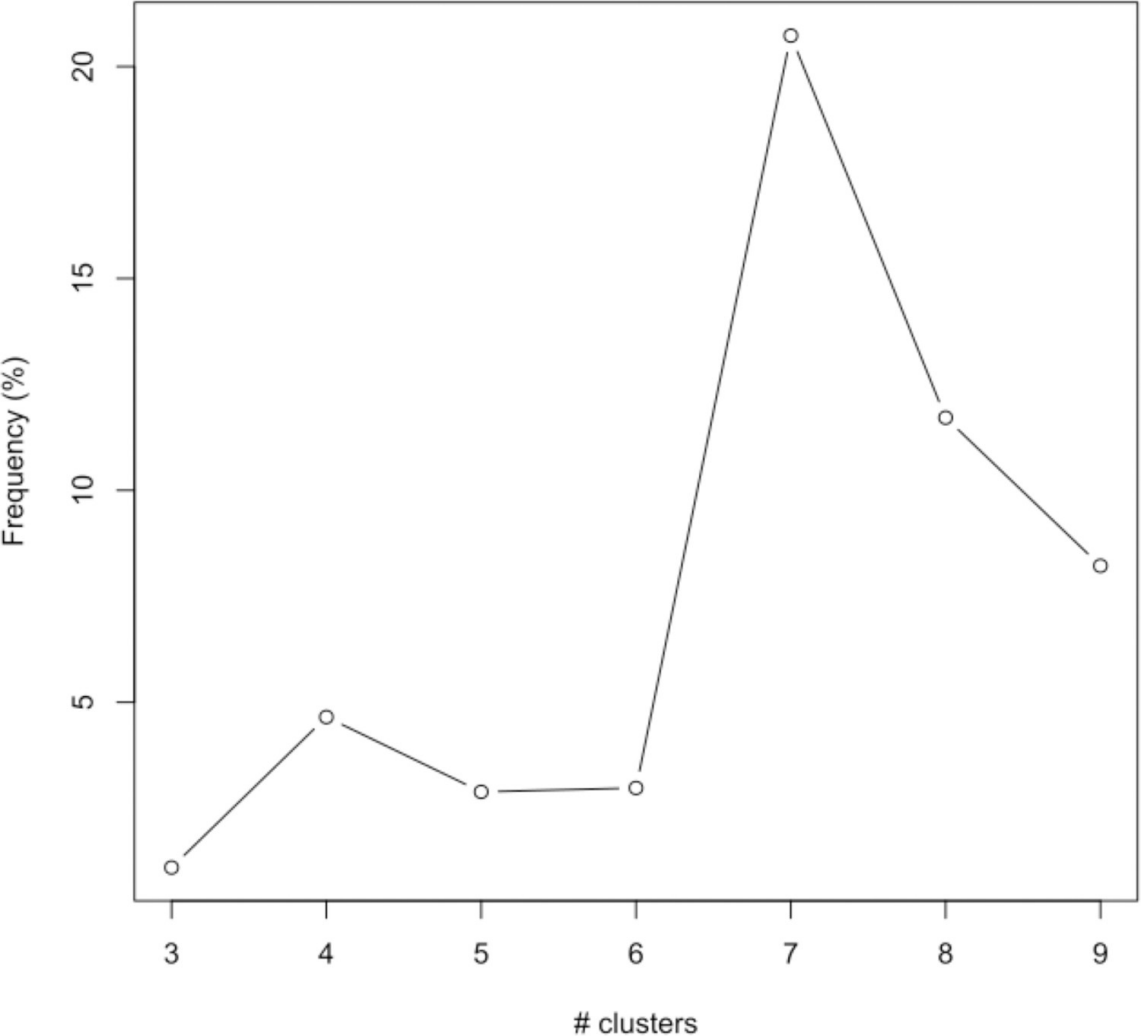
Rim data percentage for multimorbid individuals as a function of the number of clusters.

In Fig 2, the thickness of the edges between clusters reflects the proportion of individuals that have the corresponding cluster configuration, and while the structure for small cluster numbers is largely so that the structure is maintained and mainly one cluster is subdivided to form the new cluster at the bottom, there are still minor changes in allocation between the previous clusters and those that match them, indicating a presence of rim data. For example, when moving from 2 to 3 clusters, there are no such changes for individuals with at least one chronic condition, but for multimorbid individuals there are a minor number of individuals allocated to the top cluster for 2 clusters, and the middle cluster for 3 clusters. The frequency of rim data for multimorbid individuals is depicted graphically in Fig 4. Disregarding the cluster size of 3, where the within cluster sum of squares is too high to make it relevant, the frequency of rim data is smallest for 5 clusters; 2.9% of the study population for multimorbid individuals. A similar picture was observed for the population of individuals with one or more chronic conditions, also pointing to 5 clusters. Based on the elbow method, the Caliński-Harabasz index, the silhouette score and rim data presence, 5 clusters were judged to be the optimal number of clusters.

### The national multimorbidity population

Women comprised 55% of the national multimorbidity population. Mean ages for women and men were 67 years and 65 years, respectively (Table 2). The three most prevalent chronic conditions of the 16 conditions in the total population was hypertension (73.21%), hypercholesterolemia (54.99%) and allergies (26.53%), with variations between the five Danish regions (Table 1).

**Table 2:** Characteristics of the five clusters, prevalence of conditions, age and gender characteristics, educational attainment, labor market affiliation, utilization rates and cluster sizes. The Allergies cluster (cluster ALL), the Chronic Heart Conditions cluster (cluster CHC), the Hypercholesterolemia cluster (cluster CHL), the Diabetes cluster (cluster DIA), and the Musculoskeletal and Psychiatric Conditions cluster (cluster M-P).

	Cluster ALL	Cluster CHC	Cluster CHL	Cluster DIA	Cluster M-P	All
**Chronic condition (%)**						
Allergies	100.00	14.04	13.71	12.64	0.02	26.53
Anxiety	0.38	0.11	0.11	0.13	0.30	0.20
Back Pain	13.08	8.88	7.67	6.89	20.53	11.59
Cancer	7.49	9.33	8.11	6.85	18.49	10.31
CHC	5.01	100.00	0.00	0.00	1.62	18.08
COPD	26.05	19.06	12.00	10.41	23.27	18.03
Dementia	1.29	3.37	2.24	1.84	4.52	2.70
Depression	20.30	12.40	13.81	12.40	27.97	17.69
Diabetes	4.20	24.40	0.00	100.00	1.15	10.79
Hypercholesterolemia	9.25	78.89	100.00	81.98	0.08	54.99
Hypertension	44.31	76.29	86.00	83.99	71.67	73.21
Osteoarthritis	11.82	11.80	11.47	9.61	24.21	14.13
Osteoporosis	11.45	12.89	12.77	6.52	28.17	15.03
Rheumatoid Arthritis	2.02	3.23	1.67	1.92	4.75	2.71
Schizophrenia	4.53	2.21	2.16	3.45	5.73	3.58
Stroke	2.93	11.83	12.75	6.08	6.32	8.39
**Sociodemography**						
Age (years) males	58.20	69.77	67.13	64.26	64.60	65.41
Age (years) females	58.45	73.97	69.76	66.15	66.91	66.67
Age (years) all	58.36	71.50	68.57	65.11	66.09	66.12
Male frequency (%)	35.59	58.71	45.43	55.25	35.52	45.10
Female frequency (%)	64.41	41.29	54.57	44.75	64.48	54.90
**Education attainment (%)**						
No Education (≤ 10 years)	19.70	36.07	31.07	32.03	30.01	29.72
Short Education (10–14 years)	50.40	44.06	48.78	49.26	48.00	48.18
Medium Education (15–16 years)	15.89	9.61	10.70	9.90	11.34	11.50
Long Education (≥ 17 years)	11.73	6.55	7.62	5.89	7.60	7.94
Unknown Education attainment	2.28	3.71	1.85	2.92	3.06	2.66
**Labor Market Affiliation (%)**						
Employed	40.75	16.39	22.40	26.01	23.28	25.50
Unemployed	6.95	2.89	2.53	5.09	6.67	4.70
Sick leave etc.	1.10	0.46	0.40	0.67	0.99	0.71
Early retirement	13.45	9.63	10.56	14.38	13.89	12.23
Retired	34.76	69.88	63.27	52.42	53.72	55.41
Student	1.59	0.07	0.07	0.25	0.38	0.44
Unknown/Other	1.40	0.68	0.77	1.18	1.07	1.00
**Health Services Utilization (mean)**						
Hospitalizations	0.43	1.11	0.36	0.43	0.63	0.57
Bed Days	1.69	3.89	1.41	1.77	2.72	2.22
Out-patient visits	1.58	2.22	1.37	1.37	1.99	1.69
**Number of conditions**	2.64	3.89	2.84	3.45	2.39	2.97
**N, Cluster size (%)**	210.047 (18.47)	191.097 (16.81)	318.303 (27.99)	166.656 (14.66)	250.969 (22.07)	1.137.072 (100)

### Characterization of clusters by condition portfolios

The clusters identified were the **Allergies** cluster (cluster **ALL**), the **Chronic Heart Conditions** cluster (cluster **CHC**), the **Hypercholesterolemia** cluster (cluster **CHL**), the **Diabetes** cluster (cluster **DIA**), and the **Musculoskeletal and Psychiatric Conditions** cluster (cluster **M-P**). It is important to stress that the labels we imposed on the clusters constitute major and sometimes absolute trends, but the labels should not be confused with the similar conditions. For example, a person may be allocated to the Musculoskeletal and Psychiatric Conditions cluster but may have neither a musculoskeletal condition nor a psychiatric condition. Such individuals will have either cancer, COPD, or both stroke and hypertension. Similarly, a person may have the diabetes condition while not being allocated to the DIA cluster. Such individuals will be in cluster CHC if they also suffer from CHC, or in cluster ALL if they suffer from allergies but not CHC.

We characterize the clusters from the three most prevalent conditions in the cluster, conditions absent in the cluster, conditions over- or under-represented by more than 50%, and co-occurrence of the conditions. The clusters are further described by the mean number of conditions, socioeconomic variables (age, gender distribution, educational attainment, employment rate, retirement rate), and healthcare utilizations (hospitalizations, bed days, out-patient visits) and we provide a short conclusion on the cluster characteristics (Tables 2 and 3).

**Table 3:** Co-occurrences of conditions within clusters of conditions in %, in terms of dyads (2 simultaneously occurring chronic conditions), triads (3 simultaneously occurring chronic conditions) and tetrads (4 simultaneously occurring chronic conditions). The Allergies cluster (cluster ALL), the Chronic Heart Conditions cluster (cluster CHC), the Hypercholesterolemia cluster (cluster CHL), the Diabetes cluster (cluster DIA), and the Musculoskeletal and Psychiatric Conditions cluster (cluster M-P). Chronic heart conditions (CHC), chronic obstructive pulmonary disease (COPD), hypercholesterolemia (hyperchol).

**Dyad rank**	**Dyad name cluster ALL**	**Dyad pre-valence**	**Dyad name cluster CHC**	**Dyad pre-valence**	**Dyad name cluster CHL**	**Dyad pre-valence**	**Dyad name cluster DIA**	**Dyad pre-valence**	**Dyad name cluster M-P**	**Dyad pre-valence**
1	allergies, hypertension	44.31	CHC, hyperchol.	78.89	hyperchol., hypertension	86	diabetes, hypertension	83.99	hypertension, osteoporosis	18.16
2	allergies, COPD	26.05	CHC, hypertension	76.29	depression, hyperchol.	13.81	diabetes, hyperchol.	81.98	depression, hypertension	17.24
3	allergies, depression	20.3	hyperchol., hypertension	59.29	allergies, hyperchol.	13.71	hyperchol., hypertension	68.82	hypertension, osteoarthritis	15.69
4	allergies, back pain	13.08	CHC, diabetes	24.4	allergies, hypertension	13.71	allergies, diabetes	12.64	COPD, hypertension	14.79
5	allergies, osteoarthritis	11.82	diabetes, hyperchol.	21.55	hyperchol., osteoporosis	12.77	allergies, hyperchol.	12.64	cancer, hypertension	11.88
**Triad rank**	**Triad name cluster ALL**	**Triad pre-valence**	**Triad name cluster CHC**	**Triad pre-valence**	**Triad name cluster CHL**	**Triad pre-valence**	**Triad name cluster DIA**	**Triad pre-valence**	**Triad name cluster M-P**	**Triad pre-valence**
1	allergies, COPD, hypertension	8.93	CHC, hyperchol., hypertension	59.29	allergies, hyperchol., hypertension	13.71	diabetes, hyperchol., hypertension	68.82	COPD, hypertension, osteoporosis	3.14
2	allergies, depression, hypertension	6.61	CHC, diabetes, hyperchol.	21.55	depression, hyperchol., hypertension	10.42	allergies, diabetes, hyperchol.	12.64	depression, hypertension, osteoporosis	2.92
3	allergies, hypertension, osteoporosis	5.4	CHC, diabetes, hypertension	20.67	hyperchol., hypertension, osteoporosis	9.9	allergies, diabetes, hypertension	10.72	osteoarthritis, osteoporosis, hypertension	2.6
4	allergies, hypertension, osteoarthritis	5.14	diabetes, hyperchol., hypertension	18.26	hyperchol., hypertension, stroke	9.81	allergies, hyperchol., hypertension	10.72	COPD, depression, hypertension	2.39
5	allergies, back pain, hypertension	4.09	CHC, COPD, Hyperchol.	14.84	COPD, hyperchol., hypertension	9.79	depression, diabetes, hyperchol.	10.35	back pain, hypertension, osteoarthritis	2.1
**Tetrad rank**	**Tetrad name cluster ALL**	**Tetrad pre-valence**	**Tetrad name cluster CHC**	**Tetrad pre-valence**	**Tetrad name cluster CHL**	**Tetrad pre-valence**	**Tetrad name cluster DIA**	**Tetrad pre-valence**	**Tetrad name cluster M-P**	**Tetrad pre-valence**
1	allergies, COPD, depression, hypertension	1.71	CHC, diabetes, hyperchol., hypertension	18.26	allergies, COPD, hyperchol., hypertension	2.76	allergies, diabetes, hyperchol., hypertension,	10.72	COPD, depression hypertension, osteoporosis,	0.7
2	allergies, COPD, hypertension, osteoporosis	1.57	CHC, COPD, hyperchol., hypertension	11.74	allergies, depression hyperchol., hypertension	2.17	depression, diabetes, hyperchol., hypertension	8.85	back pain, hypertension, osteoarthritis, osteoporosis	0.49
3	allergies, COPD, hypertension, osteoarthritis	1.13	allergies, CHC, hyperchol., hypertension	10.7	allergies, hyperchol., hypertension, osteoarthritis	1.85	COPD, diabetes, hyperchol., hypertension	7.81	COPD, hypertension, osteoarthritis, osteoporosis	0.47
4	allergies, depression, hypertension, osteoporosis	1.09	CHC, hyperchol., hypertension, stroke	8.53	COPD, hyperchol., hypertension, osteoporosis	1.84	diabetes, hyperchol., hypertension, osteoarthritis	7.18	back pain, depression, hypertension, osteoporosis	0.46
5	allergies, back pain, depression, hypertension	1.03	CHC, depression, hyperchol., hypertension	8.29	depression, hyperchol., hypertension, stroke	1.77	diabetes, hyperchol., hypertension, osteoporosis	5.15	depression, hypertension, osteoarthritis, osteoporosis	0.45

#### Cluster ALL: The allergies cluster

Common conditions and conditions not present in the cluster: All individuals in this cluster has the condition allergies. The prevalence of hypertension, 44%, is the lowest among the clusters, while the prevalence of COPD, 26%, is the highest. Overrepresented conditions are allergies and anxiety. Underrepresented conditions are CHC, dementia, diabetes, hypercholesterolemia and stroke.

Concomitant conditions in the cluster: The 3 most common tetrads all include allergy, hypertension and COPD. Even though the prevalence of hypertension is relatively low, it plays a significant role in co-occurrence of conditions in the cluster, together with allergies. In addition, the most prevalent triad also contains COPD. With more than two conditions in this cluster, individuals will thus tend to have allergies, hypertension and COPD, and not hypercholesterolemia. All of the 5 most prevalent triads and tetrads contain allergies and hypertension (Table 3). None of the 5 most prevalent dyads, triads and tetrads include hypercholesterolemia. 15% of the individuals in this cluster have 4 or more conditions.

Mean number of conditions: The cluster has the 2^nd^ lowest number of chronic conditions (2.6).

Socioeconomic characteristics: The level of education is the highest among the clusters; 11% have a long education. The cluster is the youngest (58 years of age) and the presence of females is the 2^nd^ highest (64%). The employment rate is the highest (41%), while the rate of retired individuals is the lowest (35%).

Utilization of healthcare services: The cluster has the 2^nd^ lowest healthcare utilization rate for hospitalizations, 0.43, and bed days, 1.69. Out-patient visits are median among the clusters at 1.58.

Conclusion: The cluster is centered on individuals with allergies and associated conditions hypertension and COPD, and not related to hypercholesterolemia. The cluster appears as the least burdened, and with the highest social position.

#### Cluster CHC: The chronic heart conditions cluster

Common conditions and conditions not present in the cluster: All individuals in this cluster has CHC. The prevalence of both hypercholesterolemia (79%) and hypertension (76%) are median among the clusters. The only overrepresented condition is CHC. No conditions are underrepresented.

Concomitant conditions in the cluster: The prevalence of diabetes, 24%, is the 2^nd^ highest among the clusters. In this cluster, only the most prevalent tetrad contains diabetes, which renders diabetes as a co-condition to CHC in this cluster, and not a condition that is characteristic. Moreover, an individual with the dyad CHC and diabetes cannot be in cluster CHL nor cluster DIA, as none of these allow CHC. The cluster shows a high co-occurrence to CHC of the two conditions hypercholesterolemia and hypertension, in that 96% of the individuals in this cluster has either hypercholesterolemia or hypertension, just as all the 5 most prevalent triads contains two out of three of the conditions CHC, hypercholesterolemia and hypertension. The 5 most prevalent tetrads all contain these three conditions. The 5 most prevalent tetrads are relatively high prevalent, between 8% and 18%, indicating a concentration in the cluster around these three conditions. 56% of the individuals in the cluster have 4 or more conditions.

Mean number of conditions: The average number of conditions in this cluster is the highest among the clusters, 3.9.

Socioeconomic characteristics: The rate of education is the 2^nd^ lowest, 7% has a long education, while the individuals in the cluster are the oldest (72 years) and with the lowest presence of females, 41%. The employment rate is the lowest, 16%, while the rate of retired individuals is the highest, 70%.

Utilization of healthcare services: The healthcare utilization rate is the highest among the clusters, hospitalizations 1.11, bed days 3.89, out-patient visits 2.22.

Conclusion: The cluster concerns old people with CHC, with a high rate of males (59%), hypertension and hypercholesterolemia. The cluster is heavily burdened, both regarding the average number of conditions, social position and healthcare utilization rates.

#### Cluster CHL: The hypercholesterolemia cluster

Common conditions and conditions not present in the cluster: All individuals in the cluster have hypercholesterolemia. None of the individuals in the cluster have CHC nor diabetes. 86% have hypertension, which is the highest hypertension prevalence among the clusters. 14% of the individuals in the cluster have depression. However, while the 3^rd^ highest within cluster prevalence, this is below the national prevalence of depression at 18%. Overrepresented conditions are hypercholesterolemia and stroke. No conditions are underrepresented, apart from the absent CHC and diabetes.

Concomitant conditions in the cluster: A central point in the cluster characteristic is the dyad hypercholesterolemia and hypertension. All the 5 most prevalent triads and tetrads contain these two conditions. The condition that co-occurs most frequently with hypercholesterolemia and hypertension is allergies. All tetrads are low-prevalent and no particular tetrad stands out, which indicates that it is very variable which 4 conditions individuals has in this cluster, in case they have that many; 22% of the individuals have 4 or more conditions.

Mean number of conditions: The individuals are relatively healthy even though the number of conditions is median among the clusters (2.8), because the dominating conditions are less serious.

Socioeconomic characteristics: The level of education is the 2^nd^ highest among the clusters, 8% has a long education. The age is also 2^nd^ highest (69 years of age). The presence of females is median, 55%. The employment rate is the 2^nd^ lowest (22%), while the rate of retired individuals is the 2^nd^ highest (63%).

Utilization of healthcare services: Individuals in this cluster appear relatively healthy, having the lowest healthcare utilization rate among the clusters, hospitalizations 0.36, bed days 1.41, out-patient visits 1.37.

Conclusion: The cluster is characterized by individuals with mild conditions that generally do not burden the health care system using health services, nor appear to have serious impact on individual quality of life.

#### Cluster DIA: The diabetes cluster

Common conditions and conditions not present in the cluster: All individuals in the cluster have diabetes. The prevalence of hypertension (84%) and hypercholesterolemia (82%) are the 2^nd^ highest among the clusters, only surpassed by cluster CHL. No individuals have CHC. The only overrepresented condition is diabetes. Besides the absent CHC, underrepresented conditions are allergies and osteoporosis.

Concomitant conditions in the cluster: The co-occurrence of diabetes, hypertension and hypercholesterolemia is characteristic for the cluster, in that all individuals have either hypertension or hypercholesterolemia: Further, all the 5 most prevalent triads contain at least two out of the three conditions diabetes, hypertension and hypercholesterolemia, just as all of the 5 most prevalent tetrads contain all of these three conditions. The triad of diabetes, hypertension and hypercholesterolemia is the most prevalent triad (69%) across all clusters. The 5 most prevalent tetrads are relatively high prevalent, between 5% and 11%, indicating a concentration of tetrads in the cluster around those with diabetes, hypertension and hypercholesterolemia. Thus, with 4 conditions or more, individuals will typically have these three conditions. 41% of the individuals have 4 conditions or more in this cluster.

Mean number of conditions: The average number of chronic conditions is 3.4, which is the 2^nd^ highest among the clusters.

Socioeconomic characteristics: The level of education is the lowest among the clusters (6% has a long education). The cluster is the 2^nd^ youngest with an average age of 65 years, and has the 2^nd^ lowest presence of females, 45%. The employment rate is the 2^nd^ highest with 26%, while the rate of retired individuals is the 2^nd^ lowest with 52%.

Utilization of healthcare services: The healthcare utilization rate is generally median among the clusters, hospitalizations 0.43, bed days 1.7. However, out-patient visits are 2^nd^ lowest (1.37).

Conclusion: The cluster is centered on diabetes presence without co-occurrence of CHC, and the associated conditions hypertension and hypercholesterolemia, which nearly everyone in the cluster has. The cluster appears considerably burdened by a high number of conditions, and the lowest socioeconomic status in terms of education.

#### Cluster M-P: The musculoskeletal and psychiatric conditions cluster

Common conditions and conditions not present in the cluster: The cluster is the only cluster where no condition is completely present, just as no condition is completely absent. 72% of the individuals in the cluster have hypertension, while 28% have osteoporosis and 28% have depression. All musculoskeletal conditions, all psychiatric conditions and cancer are overrepresented. Underrepresented conditions are allergies, CHC, diabetes and hypercholesterolemia.

Concomitant conditions in the cluster: While the prevalence of hypertension is below the national average, the prevalence of osteoporosis and depression are the highest among the clusters. In fact, all musculoskeletal conditions, all psychiatric conditions (incl. dementia) and cancer show the highest prevalence in this cluster. The cluster has an overrepresentation of the co-occurrence of hypertension and osteoporosis; all the 5 most prevalent triads and tetrads contain hypertension, while 8 out of these 10 combinations also contain osteoporosis. The most common other conditions which are present among dyads, triads and tetrads are osteoarthritis and depression. Individuals in this cluster with more than two conditions thus tend to have either a musculoskeletal condition, a psychiatric condition, or both. Hypercholesterolemia is not prevalent, and does not appear at all among the 5 most prevalent dyads, triads nor tetrads. 83% of the individuals in the cluster have either a musculoskeletal condition or a psychiatric condition, while the remaining 17% have either COPD, cancer or both stroke and hypertension. Only 8% have 4 or more conditions in this cluster.

Mean number of conditions: The average number of conditions is the lowest among the clusters, 2.4.

Socioeconomic characteristics: The level of education is median (8% has a long education). Age is also median (66 years of age), while the cluster has the highest presence of females, 64%. Both the employment rate and also the rate of retired individuals are median (23% and 54%, respectively).

Utilization of healthcare services: Despite the lowest average number of conditions, individuals in the cluster have the 2^nd^ highest level of healthcare utilizations, hospitalizations 0.63, bed days 2.72, and out-patient visits 1.99.

Conclusion: The cluster is characterized by high prevalence of musculoskeletal and psychiatric conditions, while the cluster is not related to hypercholesterolemia. The cluster is burdened with the second highest healthcare utilization rate.

### Cluster formation driven by 4 conditions

The cluster formation in this analysis appears to be driven by few conditions that has a major impact. In fact, for 99.3% of the multimorbid individuals, the cluster can be determined from 4–5 conditions. In Table 4 we have listed the cluster allocation on the basis of the conditions allergies, CHC, diabetes and hypercholesterolemia. Only in the case of allergies, no CHC, no diabetes and hypercholesterolemia is it necessary to invoke the status (presence or non-presence) of hypertension as well. This is interesting, as hypertension is by far the most prevalent condition, but it is not one of the 4 primary conditions that drive the cluster formation.

**Table 4:** Cluster allocation for individuals based on 4–5 conditions. Applies to 1.129.342 out of 1.137.072 individuals (99.32%). The Allergies cluster (cluster ALL), the Chronic Heart Conditions cluster (cluster CHC), the Hypercholesterolemia cluster (cluster CHL), the Diabetes cluster (cluster DIA), and the Musculoskeletal and Psychiatric Conditions cluster (cluster M-P).

Allergies	CHC	Diabetes	Hypercholesterolemia	Cluster
NO	NO	NO	NO	**M-P**
YES	NO	NO	NO	**ALL**
NO	YES	NO	NO	**CHC**
NO	NO	YES	NO	**DIA**
NO	NO	NO	YES	**CHL**
YES	YES	NO	NO	**ALL**
YES	NO	YES	NO	**ALL**
YES	NO	NO	YES	Hypertension YES: **CHL** Hypertension NO: **ALL**
NO	YES	YES	NO	**CHC**
NO	YES	NO	YES	**CHC**
NO	NO	YES	YES	**DIA**
YES	YES	YES	NO	**CHC**
YES	YES	NO	YES	**CHC**
YES	NO	YES	YES	**DIA**
NO	YES	YES	YES	**CHC**
YES	YES	YES	YES	**CHC**

### Single condition individuals in the cluster analysis for individuals with at least one chronic condition

The supplementary cluster analyses for individuals with at least one chronic condition pointed towards 5 clusters, similarly to the analysis for multimorbidity individuals. In the population of individuals with chronic conditions, 958.457 individuals only had a single condition. However, in the supplementary 5 cluster analysis, single condition individuals were all contained in the same cluster, except for the conditions hypertension, hypercholesterolemia and allergies. Single condition individuals with any of these three conditions were contained in three other distinct clusters. The inclusion of single condition individuals thus leaves little extra information.

### Distribution of the 5 clusters in the five regions of Denmark

**Fig 5:**
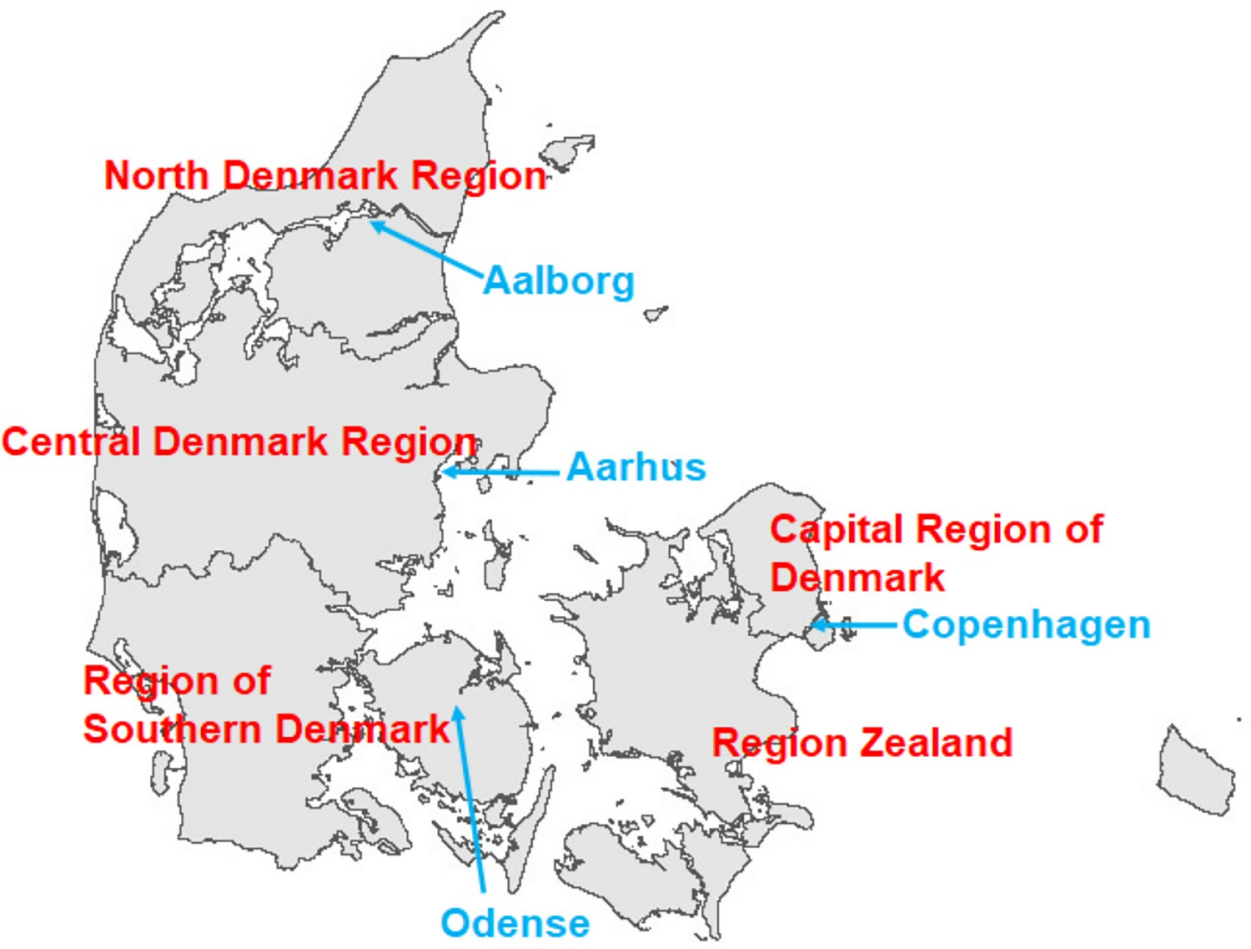
The Danish regions (in red), and cities with more than 100.000 inhabitants (in turquoise). Figure utilizes geographical information from Agency for Data Supply and Infrastructure, regional borders September 2023, https://dataforsyningen.dk/data/4838.

The five Danish Regions are depicted in Fig 5. Cluster frequencies among the Danish regions are listed in Table 5. Cluster CHL shows the highest rate in the North Denmark Region (31.13%). Cluster M-P shows the highest rates in the Region of Southern Denmark (23.98%) followed by Region Zealand (21.87%). The Allergies cluster shows the highest rates in the Capital Region of Denmark (20.85%) and shows the highest rates in east Denmark. Cluster CHC show the highest rate in Region Zealand (18.04%) followed by the Central Denmark Region (17.44%). Cluster DIA shows the highest rates in east Denmark; Region Zealand (15.56%) and the Capital Region of Denmark (15.00%).

**Table 5:** Cluster prevalence at the national level and in the five Danish regions. The Allergies cluster (cluster ALL), the Chronic Heart Conditions cluster (cluster CHC), the Hypercholesterolemia cluster (cluster CHL), the Diabetes cluster (cluster DIA), and the Musculoskeletal and Psychiatric Conditions cluster (cluster M-P).

Region	Multimorbidity population (N)	Cluster ALL (%)	Cluster CHC (%)	Cluster CHL (%)	Cluster DIA (%)	Cluster M-P (%)
Capital Region of Denmark	307.880	20.85	17.12	25.75	15.00	21.27
Central Denmark Region	256.566	17.92	17.44	28.93	14.02	21.69
North Denmark Region	126.733	16.95	16.05	31.13	14.89	20.99
Region of Southern Denmark	269.597	17.06	15.39	29.39	14.17	23.98
Region Zealand	176.296	18.38	18.04	26.15	15.56	21.87
**Denmark**	1.137.072	18.47	16.81	27.99	14.66	22.07

## Discussion

### Main findings

The study population comprised 4.489.821 individuals, of which 958.457 had one chronic condition and 1.137.072 had multimorbidity. Considering the multimorbidity population, the population included a higher proportion of women, 54.9%. Women were older than men, mean ages were 66.7 years and 65.4 years respectively, which is in alignment with earlier findings [35]. 16 chronic conditions were registered for the multimorbidity population.

Five multimorbidity clusters were identified using the K-means method; The Allergies, Diabetes and Chronic Heart Conditions, Hypercholesterolemia and Musculoskeletal and Psychiatric Conditions clusters. The clusters were characterized by the three most prevalent conditions in the cluster, and also conditions that were not in the cluster. For 4 out of 5 clusters, all individuals in the cluster had a chronic condition that gave name to and characterized the cluster. The remaining cluster, the Musculoskeletal and Psychiatric Conditions cluster, was characterized by the presence of musculoskeletal and/or psychiatric conditions.

### Characteristics of clusters

A criterion for cluster allocation should be robust, and should not depend on whether a new cluster is added or not, in the sense described in the methods section. This prompted us to use a low frequency of rim data to indicate high robustness in cluster allocation, as a high frequency of rim data signifies increased erratic cluster allocation, which is undesirable, as one among several statistics when determining the number of clusters.

Including single condition individuals in the cluster analysis did not result in enough additional information to justify it.

The five clusters identified, the Allergies, Chronic Heart Condition, Diabetes, Hypercholesterolemia, and Musculoskeletal and Psychiatric Conditions clusters, were further characterized by the cluster population distributions of gender, age, educational attainment, employment and retirement rates, and healthcare utilization patterns, and showed varying patterns for those variables.

The cluster with the on average youngest individuals is the Allergies cluster (58.36 years), followed by the Diabetes cluster (65.11 years). Both clusters are characterized by a high prevalence rate of conditions that is less serious and with a low disease burden. The Chronic Heart Conditions cluster includes the oldest individuals (71.50 years) followed by the Hypercholesterolemia cluster (68.57 years).

The cluster with highest rates of women is the Musculoskeletal and Psychiatric Conditions cluster (64.48%), followed by the Allergies cluster (64.41%). The most prevalent musculoskeletal or psychiatric condition in the cluster is osteoporosis. Osteoporosis is a gender specific condition characterized by high prevalence rates in women [36], increasing with higher age. Female individuals are also suffering from specific chronic conditions such as COPD, which shows the highest prevalence in these two clusters. Depression is also known to appear with high prevalence in women [37], and has the highest prevalence in these two clusters.

The cluster with the highest rate of men is the Chronic Heart Conditions cluster (58.71%), followed by the Diabetes cluster (55.25%). Both corresponding chronic conditions, CHC and diabetes, are associated with higher prevalence rates in men [38, 39].

The Hypercholesterolemia cluster has the lowest healthcare utilization rate (hospitalizations 0.36, bed days 1.41, out-patient visits 1.37). The most prevalent chronic conditions in the cluster are hypercholesterolemia and hypertension, and the individuals are relatively healthy. The Hypercholesterolemia cluster is followed by the Allergies cluster. Individuals suffering from conditions in those two clusters do mostly not experience difficult symptoms, and mostly need subscription medicine. Regarding individuals with hypercholesterolemia, they mostly need regularly control visits with their GP, or in an out-patient clinic. The cluster with the highest healthcare utilization rates is the Chronic Heart Conditions cluster (hospitalizations 1.11, bed days 3.89, out-patient visits 2.22), which can be explained both by the highest mean age of the individuals in the cluster, but also by that individuals with CHC are often treated by complex medicine schemes that often demand frequent regulation. Further, when the conditions develop over time, the individual often need regular out-patient visits, and sometimes also hospitalizations to stabilize the CHC aggravation over time [40]. The Musculoskeletal and Psychiatric Conditions cluster also show high utilization rates (hospitalizations 0.62, bed days 2.7, out-patient visits 1.99), which we ascribe to the high rates of both the musculoskeletal conditions and especially depression in the cluster [41].

### Cluster patterns of conditions in other studies

This study aims to describe the most common concurrent chronic conditions from disease clusters in a national multimorbidity population. The purpose is in part to explore the possibility for supporting the population management, and possibly the development of clinical guidelines for the most common concurrent chronic conditions in people with multimorbidity. A systematic review for clinical applications of population stratification or segmentation [42] concluded that methods for segmentation of populations hold great potential for population management, as for example to develop and organize care based on different care programs tailored for various segments, and thereby provide more effective healthcare planning and evidence-based care. The focus of this systematic review is much in line with the aim of this present study.

A literature review based on 39 articles report patterns of multimorbidity in primary care [7]. The definition of multimorbidity and diagnosis classification systems in the studies varied. While 24 of their studies reported information on multimorbidity patterns, the majority focused on descriptive information on two to three co-occurring conditions only. The most frequent conditions constituting the patterns of multimorbidity were hypertension and osteoarthritis, followed by combinations of cardiovascular conditions. Only 6 of the studies reported having performed statistical cluster analysis or factor analysis, and among those performing cluster analysis the authors reported no consistent pattern. The review study indicates a lack of standards for studying patterns of multimorbidity that we believe still persists. A similar claim is made in [43].

A Danish study reported seven classes of individuals, labeled; 1. Relatively healthy, 2. Hypertension, 3. Musculoskeletal disorders, 4. Headache-mental disorders, 5. Asthma-allergy disorders, 6. Complex cardio metabolic disorders and 7. Complex respiratory disorders, from a Danish population including 162.283 individuals older than 16 years [44]. The study was repeated in 2021, and the clusters identified there were similar to those in the study from 2017 [45]. The study used the Latent Class Analysis (LCA) statistical method, and the population comprised a randomized sample of the Danish population. The nature of the statistical model-based LCA method is very different from the data driven K-means methods applied here. LCA results in “classes”; a latent structure resembling clusters. The standard LCA method requires that also individuals with 0 and 1 chronic condition are included in the study population to function appropriately. We note a minor overlap in cluster labels with ours, but also differences in study populations. We only used multimorbid individuals for our cluster analyses, and for the decision on the number of clusters. While K-means and LCA results may still be linked, the logic and relevance of the linking is not immediate. We are pursuing comparisons of the LCA and the K-means methods in forthcoming research.

A Spanish study found 6 multimorbidity patterns, five related to organ systems and one nonspecific. The study included a primary care population of 24.013 individuals between 65–94 years of age [46]. This study used Multiple Correspondence Analysis followed by K-means clustering, on data stratified by age and gender. The data was confined to urban data from the city of Barcelona. This, and the advanced age of the study population, makes comparisons to our work difficult; however, we note that the authors arrived at a similar number of clusters as we do.

### Statistical methods used for identification of clusters

Methodologies for cluster identification has been subject to much debate [47, 48]. In [42] it is reported that the authors find that the appropriate methodology to classify populations, rather than conditions, is clustering techniques, while factor analysis is reported as an appropriate methodology for classifying conditions. This conforms to our analysis. In [42], the most common cluster analysis methods for human chronic conditions data was LCA, followed by K-means and Hierarchical Clustering. For the data that we wanted to study, the standard LCA method is ill suited due to the requirement of individuals being allowed to have 0 or 1 chronic condition. Hierarchical clustering is computationally intensive for large datasets, and with that in mind we decided to use the data driven K-means method. A similar view is taken in the study [49], published by the same research group as [43]. The group found that non-hierarchical clustering provided ‘an informative categorization of patients, generating reasonable multimorbidity patterns from a clinical, practical perspective’, and identified ‘phenotypes for sub-groups of patients. While the method obviously depends on the number of chronic conditions considered, we have earlier performed comparative studies of chronic conditions in populations with 16 and 42 conditions respectively [3, 4]. In these studies it appears that the overall patterns of numbers of chronic diseases are similar even when considered age-dependent, which we ascribe to that the main drivers of chronic conditions are present in both collections of chronic conditions. We thus expect our results to relate to populations and health sectors similar to the Danish, essentially irrespective of the conditions considered.

We found that for nearly the entire multimorbidity population, the cluster allocation could be determined by the presence or non-presence for 4–5 conditions (Table 4). We stress that the conditions appearing in Table 4 refers to the multimorbid part of the population. When clustering other types of populations of individuals (eg. the full adult population), we have found that other conditions enter as determinators for cluster allocation.

### Spatial variation of cluster frequencies

The spatial distribution according to the Danish Regions is listed in Table 5. Some of the clusters appear to be correlated with socioeconomic status, measured through the frequency of individuals with a long education. The Danish public sector is divided into 98 municipalities and 5 regions. To assess the correlation with socioeconomic status, we calculated the cluster frequencies at municipality level, and regressed cluster frequencies on the frequency of multimorbid individuals that were registered with a “long education”, similar to the national numbers appearing in Table 2. The analysis revealed that cluster ALL was positively correlated with socioeconomic status represented this way, while clusters CHL and DIA were negatively correlated with socioeconomic status. However, we did not detect any correlation to socioeconomic status for cluster M-P and cluster CHC. A similar pattern, but less stringent, appeared when we represented socioeconomic status with the frequency of individuals registered with “no education”, in Denmark equal to primary school or less, up to 10 years of education.

**Fig 6:**
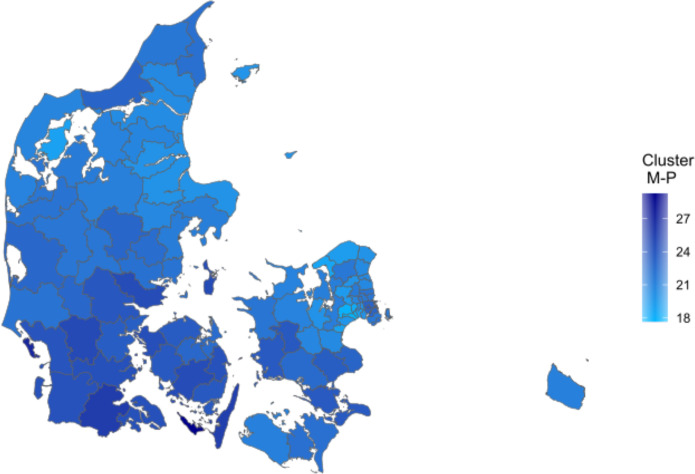
Spatial variation in the Musculoskeletal and psychiatric conditions cluster. Cluster prevalence in %. Figure utilizes geographical information from Agency for Data Supply and Infrastructure, municipality borders September 2023, https://dataforsyningen.dk/data/3901.

While cluster M-P does not seem to correlate with socioeconomic status, it is still spatially heterogeneous. In Fig 6, Denmark is depicted at the municipality level, with municipality shapes filled according to the prevalence of cluster M-P. From the color coding it is clear that the southern part of Denmark (not to be confused with the Region of Southern Denmark, Fig 5) has a considerably higher prevalence than the northern part of Denmark. We do not at present have an explanation for this geographical heterogeneity.

**Fig 7:**
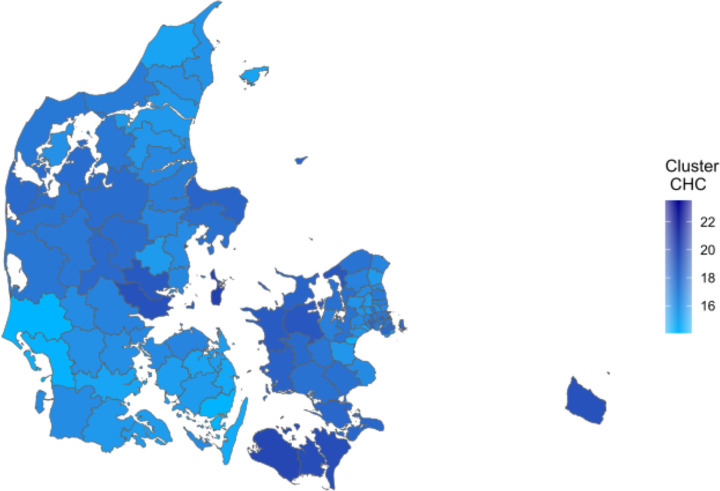
Spatial variation in the chronic heart conditions cluster. Cluster prevalence in %. Figure utilizes geographical information from Agency for Data Supply and Infrastructure, municipality borders September 2023, https://dataforsyningen.dk/data/3901.

Similarly, we depicted the prevalence of Cluster CHC in Fig 7. It is clear from Fig 7 that cluster CHC is highly prevalent in the western part of the Central Denmark Region, and in the western and southern parts of Region Zealand. These are areas with a low degree of urbanization, and the cluster has low prevalence in the municipalities that contain the 4 cities in Denmark with more than 100.000 inhabitants: Copenhagen, Aarhus, Odense and Aalborg (Fig 5). However, we do not have access to relevant geographical information that would allow us to investigate if the degree of urbanization is indeed correlated with cluster CHC prevalence.

### A further observation

It is striking that cancer, despite a relatively high prevalence, plays little to no role in cluster formation, exemplified in that cancer only appears in 1 out of 75 prevalence top-5 of within-cluster dyads, triads and tetrads. We hypothesize that this may be caused by that the algorithmic diagnosis cancer is a meta-diagnosis, in the sense that it is a collective term for a wide range of conditions, that aren’t confined to a specific organ. As such, the pathologies of cancer conditions may not by themselves lead to specific comorbidities, but these may be acquired through association, that while correlated with the cancer (t.ex. depression) will not be rooted in the pathology. For a similar discussion, see [50].

## Strengths and limitations

The major strength of our study is the use of algorithmic diagnoses, which allows us to include the entire Danish population and obviates the need for considerations on t. ex. Representativity. An additional strength is the large scale of the study with inclusion of comprehensive information about chronic conditions, sociodemographic information and healthcare utilization. In general, the Danish national registers provide full information about healthcare system contacts. The registers maintain high quality and reliability, and they are well suited and used extensively for research [51]. Being a full population based study, our findings reflect the real-world situation, where uncertainty is limited to the accuracy of the algorithmic diagnoses [28]. The use of full population register data finds our study free of eg. recall bias and loss to follow up, and disturbances such as sampling variation.

The necessary use of diagnostic algorithms to identify individuals with chronic conditions in the primary healthcare sector is an approximation of actual diagnoses. Although the diagnostic algorithms have been shown to be highly accurate [28], they are not true diagnoses in the sense that they are not clinically determined by physicians, nor will they diagnose conditions for individuals that do not contact their GP. This and the cross-sectional form of our data, collected during a single year, points towards the risk of underestimation of the number of individuals with specific chronic conditions [52]. [44] contains studies of a representative survey from the Danish population in 2013, which in structure is similar to a representative subset of our data, although two years earlier. In this study, the prevalence of a condition that relies solely on ICD-10 codes, cancer, was reported at 3%. This conforms with our diagnostic algorithms, when including individuals without multimorbidity. However, other conditions appear at a higher prevalence, which we exemplify in allergies and arthritis. Allergies were reported at 21%, even when disregarding asthma (7%), which is nearly double compared to our data (12%). Arthritis was reported at 21% as well. When pooling rheumatoid arthritis and osteoarthritis in our diagnostic algorithms, this number is four times as high as our prevalence of 5%, again including individuals without multimorbidity. We expect that these three prevalences reflect that the diagnoses in [44] are self-reported, and thus composed of individuals with similar diagnoses when applying diagnostic algorithms, individuals that incorrectly report the condition from misperceptions etc., and individuals that correctly report the condition but will not have been in contact with the health authorities, and thus will not be caught by the algorithmic diagnoses. For individuals that have cancer, very few will not be in contact with the health authorities, and there will be few that incorrectly report this condition. Thus, it is to be expected that the survey agrees with the diagnostic algorithms. However, for both allergies and arthritis, we hypothesize that the increased prevalences in [44] reflects lack of clarity of the perceived definition of the condition, causing individuals to incorrectly report the condition, and that individuals suffering from any of these two conditions may not have been in contact with the health authorities, due to eg. lack of severeness of the condition. Both reasons for reporting will increase the survey prevalence, which is of a different magnitude than the prevalence obtained from algorithmic diagnoses.

3% of our study population did not have information on educational attainment (Table 2). This information appeared to be missing at random, except for individuals aged 94 years and above. Personal communication with Statistics Denmark has uncovered that Danish administrative registers only contain information on education for individuals born after 1920, which we believe is the cause of this. However, this group only contained 0.8% of the multimorbid individuals, and we estimated the effect in frequency estimation (Table 2) to be very small.

Comparisons of studies on multimorbidity in populations should be performed with care. Studies may differ in definitions of multimorbidity (i.e., two or more chronic conditions in this study), included conditions which the multimorbidity refers to, data collection methods, characteristics of the populations, important factors for risk of development of chronic conditions and multimorbidity such as age, gender and socioeconomic status, rendering comparisons difficult. However, challenges on included conditions and data collection methods may be overcome for large studies [4].

## Conclusion

Five clusters were identified using the K-means cluster analysis for the multimorbidity population in Denmark in 2015, based on 16 chronic conditions. Each of the clusters comprised about the same number of individuals. Four conditions were important for the cluster determination. The identification of the clusters give rise to new knowledge on the co-occurrence of chronic conditions in the Danish population, and has the potential for clinical applications, such as supporting improved health care provision in individuals with multimorbidity and the development of multimorbidity clinical guidelines for simultaneously occurring conditions, which may among other things reduce commonly occurring problems such as cross-medication. Further, information regarding specific clustering might be supportive for population management. The use of rim data for selection of clusters is a stability criterion that is found prior to a decision on the number of clusters and will be pursued in further research.

## Supporting information

S1 Table Algorithmic diagnoses. Algorithms used to define the 16 algorithmic diagnoses utilizing the Danish National Patient Register (NPR), the Danish Psychiatric Central Research Register (PCRR) and the Danish National Prescription Registry (DNPR). Algorithmic diagnoses are evaluated per January 1^st^ 2015.
(DOCX)
