# Correction: Clusters from chronic conditions in the Danish adult population

**DOI:** 10.1371/journal.pone.0324458

**Published:** 2025-05-12

**Authors:** Anders Stockmarr, Anne Frølich

The Data Availability statement for this article is incorrect. The correct statement is: Due to restrictions related to Danish law and protecting patient privacy, the combined set of data as used in this study can only be made available through a trusted third party, Statistics Denmark. This state organisation holds the data used for this study. Danish scientific organisations can be authorized to work with data within Statistics Denmark and such organisations can provide access to individual scientists inside and outside of Denmark. Requests for data may be sent to Statistics Denmark: http://www.dst.dk/en/OmDS/organisation/.
