# Supplementary material for: Clusters from chronic conditions in the Danish adult population
Source: PLoS One. 2024 Apr 30;19(4):e0302535. doi: 10.1371/journal.pone.0302535 (PMC11060538; doi:10.1371/journal.pone.0302535)
Supplement: S1 Table — Algorithms used to define the 16 algorithmic diagnoses utilizing the Danish National Patient Register (NPR), the Danish Psychiatric Central Research Register (PCRR) and the Danish National Prescription Registry (DNPR). Algorithmic diagnoses are evaluated per January 1st 2015. (DOCX) [file pone.0302535.s001.docx]

Supplemental Table S1. Algorithmic diagnoses. Algorithms used to define the 16 algorithmic diagnoses utilizing the Danish National Patient Register (NPR), the Danish Psychiatric Central Research Register (PCRR) and the Danish National Prescription Registry (DNPR). Algorithmic diagnoses are evaluated for the year 2015.

| Chronic condition | ICD-10 from NPR and PCRR | Definition |
| --- | --- | --- |
| Allergies | J30.1-J30.9 | (DIAG)*^a^* and/or (MEDICINE)*^b^* with ATC: V01AA11. V01AA02, V01AA03, V01AA05, R01AC, R01AD, R06A, S01G, R01BA52.  Two prescriptions within a period of two years. |
| Anxiety | F40.1, F41.1 | (DIAG)*^a^* and (MEDICINE)*^b^* with ATC: N06A. Note: At least three different prescriptions with at least 2 years between the first and last one. Patients with depression are excluded from this algorithm. |
| Back pain | M40-M54 | (DIAG)*^a^* |
| Cancer | C00-C43, C45-C97 | (DIAG)*^a^* |
| Chronic Heart Condition | I20, I21, I23-I25, I50, I11, I13 | (DIAG)*^a^* and/or (MEDICINE)*^b^* with ATC: C01A,  C01B, C01D, C01E |
| Chronic obstructive  pulmonary disease  (COPD) | J40-J44, J47, J96 | (DIAG)*^a^* All patients aged 35 years or older at contact. And/or (MEDICINE)*^b^* with ATC: V03AN01 if they do not have contacts with ICD-10 codes J45 or J46, also aged 35 years or older at the date of distribution. And/or (SERVICE)*^c^* with lab services 807113 (lung spirometer test), 807121 (lung function test), also aged 35 years or older at the time of service. |
| Dementia | F00, G30, F01, F02.0, F03.9,  G31.8B, G31.8E, G31.9, G31.0B | (DIAG)*^a^* All patients aged 60 years or older at contact. And/or (MEDICINE)*^b^* with ATC: N06D, also aged 35 years or older at the date of distribution. |
| Long term use  of antidepressants  (Depression) |  | (MEDICINE)*^b^* with ATC: N06A. Note: At least three different prescriptions with at least 2 years between the first and last one. Patients with schizophrenia or dementia are excluded from this algorithm. |
| Diabetes | E10-E14, H28.0, H36.0 | (DIAG)*^a^* and/or (MEDICINE)*^b^* with ATC: A10. A10BA02 is excluded for females aged 20-40 years at the date of distribution |
| Hypercholesterolemia | E78.0, E78.2, E78.4, E78.5 | (DIAG)*^a^* and/or (MEDICINE)*^b^* with ATC: C10 |
| Hypertension | I10-I13, I15 | (DIAG)*^a^* and/or (MEDICINE)*^b^* with ATC: C07B, C03A, C03B, C03E, C03X and/or (MEDICINE)*^b^* with ATC: C03C, C03D, C07A, C09 if they do not have contacts with ICD-10 codes I20.0, I21, I25.1, I50 and/or (MEDICINE)*^b^* with ATC: C08 if they do not have contacts with ICD-10 codes I20-25 |
| Osteoarthritis | M15-M19 | (DIAG)*^a^* |
| Osteoporosis | M80-M82 and/or for persons aged 45 years or older S22.0, S22.1, S32.0, S32.7, S32.8, S42.2, S42.4,  S42.7-S42.9, S52.5-S52.9, S62.0,  S62.1, S72 | (DIAG)*^a^* and/or (MEDICINE)*^b^* with ATC: M05B,  G03XC01, H05AA02, H05AA03 |
| Rheumatoid Arthritis | M05, M06.0, M06.8, M07.0, M07.1, M10.0, M10.9 | (DIAG)*^a^* |
| Schizophrenia | F20-F22, F25, F28, F29, F31 | (DIAG)*^a^* and/or (MEDICINE)*^b^* N05AX13, N05AX12,  N05AH03, N05AX08 |
| Stroke | G45, G46, I60-I69 | (DIAG)*^a^* |

*^a^* DIAG: All patients at any age unless otherwise specified, who had a hospital inpatient or outpatient encounter with one of the ICD-10 codes specified in the column ICD-10 from NPR and PCRR in the past five years. Primary (A) and secondary (B) diagnoses are considered.

*^b^* MEDICINE: All patients at any age unless otherwise specified, who in the past five years had a minimum of two medicine prescriptions from DNPR on two separate days with the stated ATC codes within a period of one year.

*^c^* SERVICE: All patients who within the past five years had a minimum of two healthcare services from NHSR with either of the stated lab service codes on two separate days within a period of one year.
